# Neural correlates of musical timbre: an ALE meta-analysis of neuroimaging data

**DOI:** 10.3389/fnins.2024.1373232

**Published:** 2024-06-17

**Authors:** Oliver Tab Bellmann, Rie Asano

**Affiliations:** ^1^Acoustics Research Institute, Austrian Academy of Sciences, Vienna, Austria; ^2^Systematic Musicology, Institute for Musicology, University of Cologne, Cologne, Germany

**Keywords:** timbre, ALE meta-analysis, dual-stream model, music cognition, cortical processing

## Abstract

Timbre is a central aspect of music that allows listeners to identify musical sounds and conveys musical emotion, but also allows for the recognition of actions and is an important structuring property of music. The former functions are known to be implemented in a ventral auditory stream in processing musical timbre. While the latter functions are commonly attributed to areas in a dorsal auditory processing stream in other musical domains, its involvement in musical timbre processing is so far unknown. To investigate if musical timbre processing involves both dorsal and ventral auditory pathways, we carried out an activation likelihood estimation (ALE) meta-analysis of 18 experiments from 17 published neuroimaging studies on musical timbre perception. We identified consistent activations in Brodmann areas (BA) 41, 42, and 22 in the bilateral transverse temporal gyri, the posterior superior temporal gyri and planum temporale, in BA 40 of the bilateral inferior parietal lobe, in BA 13 in the bilateral posterior Insula, and in BA 13 and 22 in the right anterior insula and superior temporal gyrus. The vast majority of the identified regions are associated with the dorsal and ventral auditory processing streams. We therefore propose to frame the processing of musical timbre in a dual-stream model. Moreover, the regions activated in processing timbre show similarities to the brain regions involved in processing several other fundamental aspects of music, indicating possible shared neural bases of musical timbre and other musical domains.

## 1 Introduction

Music cognition research explores the neural bases of processing essential features of music, including pitch and rhythm. These features have been the primary focus within a dual-stream model. Consistent with visual processing streams (Goodale and Milner, 1992), two auditory processing streams were introduced: the auditory dorsal stream, connecting the areas in the posterior superior temporal gyrus, the inferior parietal lobes, premotor areas, and posterior parts of the inferior frontal gyrus, is considered to be involved in sequencing, whereas a ventral stream, linking the anterior superior temporal gyrus and anterior areas of the inferior frontal gyrus, is conceived of playing central role in categorical perception and auditory pattern recognition (Zatorre et al., 2007; Rauschecker, 2014). For example, concerning pitch processing, the areas related to the auditory dorsal streams are involved in the processing of interval structures (Stewart et al., 2008), the concatenation of pitches into a sequence (Green et al., 2018), the syntactic processing of chord progressions (Musso et al., 2015), while those associated with the ventral stream deal with the mapping of semantic and affective information onto pitch sequences (Rauschecker, 2014; Asano et al., 2022b). Concerning rhythm, the areas related to the dorsal auditory stream are involved in the timing (Grahn et al., 2011), sequencing (Chen et al., 2008; Kotz et al., 2018), and metrical structuring of events (Chen et al., 2008), the organization of sounds into beat patterns (Patel and Iversen, 2014), and the production of rhythms (Zatorre et al., 2007; Kotz et al., 2018), while the ventral stream areas are involved in the integration of melodic and rhythmic information (Sihvonen et al., 2022).

Another essential feature of music besides pitch and rhythm is timbre (Patel, 2008; Holmes, 2012; Hoeschele et al., 2014). Timbre, also called “sound quality” or “tone color”, is a perceptual property of sounds, which enables listeners to discriminate two sounds equal in all other parameters such as pitch, duration, and loudness (Siedenburg et al., 2016). For example, timbre allows listeners to distinguish a note plucked on a violin vs. a guitar; a violin sound plucked vs. bowed; or to discriminate the elements in the sound sequence “/p, /t, /k, /t,” when all other parameters are kept constant (Koelsch et al., 2002; Town and Bizley, 2013; Siedenburg et al., 2016). Timbre depends on multiple acoustic properties, such as the attack portion, the composition of the frequency spectrum, and the development of the spectrum in time (McAdams, 2013).

The ability to perceive timbre is considered to be a feature of human musicality (Wagner and Hoeschele, 2022). Already infants remember the timbre of unfamiliar songs better than their melody (Trainor et al., 2004), and changes in timbre considerably influence the recognition of music by adults (e.g., Radvansky et al., 1995; Lange and Czernochowski, 2013; Schellenberg and Habashi, 2015). Timbre is a quality inherent to all perceived musical instrument sounds and vocal sounds (Town and Bizley, 2013). It is fundamental for the discrimination of sounds, the identification and recognition of sound sources such as musical instruments (Handel, 1995), and the categorization of actions involved in, e.g., various types of drum strokes (Patel, 2008). Timbre is also seen as one of the primary ways of conveying emotion in music (Hailstone et al., 2009; Holmes, 2012; Bowman and Yamauchi, 2016; Juslin and Lindström, 2016).

Like pitch and rhythm, timbre is also an important structuring property of music (Patel, 2008; Goodchild et al., 2019; McAdams, 2019). As timbre allows the discrimination of sound events that occur in temporal succession (Town and Bizley, 2013; Koelsch, 2019), it has repeatedly been proposed to enable the segmental grouping of sounds into larger musical units (McAdams and Giordano, 2016; Goodchild et al., 2019; McAdams, 2019). For example, percussive and beatboxing music can be essentially regarded as a structured series of timbres, in which the musical patterns are organized based on sound events that mainly differ in timbre (Patel, 2008; Stowell and Plumbley, 2008). Moreover, repeating sequences with hierarchical dependencies among timbres are recognized even by listeners unfamiliar with these patterns (Siedenburg et al., 2016).

Previous research found that musical timbre processing is commonly attributed to the areas related to the ventral stream including the middle and anterior portions of the superior temporal gyrus (STG) and the ventral part of the inferior frontal gyrus (IFG) (Rauschecker, 2014; Alluri and Kadiri, 2019). Timbre-based analyses of basic auditory features (such as the perceived “brightness”) and discrimination of sound events are thought to be mediated by central auditory cortex (AC) and directly adjacent cortices of the posterior STG (pSTG), including its upper surface posterior to the primary auditory cortex (planum temporale, PT), while stimulus categorization based on timbre is thought to be mediated by cortices in the anterior STG (aSTG) including its superior surface (planum polare, PP) anterior to the primary auditory cortex (Town and Bizley, 2013; Alluri and Kadiri, 2019; Wei et al., 2022). It was also suggested that musical timbre processing mainly depends on right-hemispheric structures (Samson et al., 2002).

To date, the contributions of the dorsal stream to (musical) timbre processing remain unclear (Alluri and Kadiri, 2019). One of the central functions of the dorsal auditory stream in music processing is the processing of pitch and rhythmic sequences. For example, in processing melodies, the dorsal stream is associated with the discrimination of pitches and the sequencing of pitch patterns in the pSTG, PT, IPL, PMC, and SMA (Rauschecker and Scott, 2009; Rauschecker, 2014) as well as the structural organization of pitch and the prediction and detection of deviations from pitch patterns in the PT, pSTG, and IFG (Zatorre et al., 2007). Likewise, timbre processing involves these operations typically implemented in the dorsal stream: Based on timbre, sounds are discriminated in a continuous stream of incoming information and organized in a specific order, which is crucial for the structuring of sequences from multiple sounds (Patel, 2008; McAdams, 2013). Moreover, on the basis of timbre, listeners can learn structural regularities of musical sequences (Tillmann and McAdams, 2004) and detect deviations from a sequence of sounds (Koelsch et al., 2002). Therefore, we hypothesize that musical timbre processing involves areas in the dorsal auditory stream. To test this hypothesis, we conduct an activation-likelihood estimation (ALE) meta-analysis on the neural correlates of musical timbre perception. Although two ALE meta-analyses on musical timbre processing have been already conducted before (Janata, 2015; Criscuolo et al., 2021), these analyses provide no clear answers regarding the cortical organization of musical timbre processing due to small sample sizes, inconsistency in study inclusion, and/or inclusion of non-musical stimuli.

Based on the previous research, we expect to identify consistent activations in (1) auditory regions on the HG (Ba 41, BA 42) that perform basic timbral analyses (Janata, 2015; Alluri and Kadiri, 2019) and (2) in ventral stream areas aSTG/PP (BA 22) for processing categorical information for object recognition (Bizley and Cohen, 2013; Janata, 2015). With timbre as a structuring property involved in segmentation, we also expect (3) dorsal stream associated activations in pSTG/PT (BA 22) (Janata, 2015) for timbre-based discrimination (Town and Bizley, 2013) and sequential processing (Clark et al., 2015). We also expect the inferior parietal lobe (IPL) and the IFG (Criscuolo et al., 2021), which were associated with musical timbre-based sensorimotor, sequential (BA 39 & BA 40; Margulis et al., 2009; Lévêque and Schön, 2015), and structural (BA 44; Koelsch et al., 2002; Koelsch, 2006) processing.

## 2 Method

**Figure 1:**
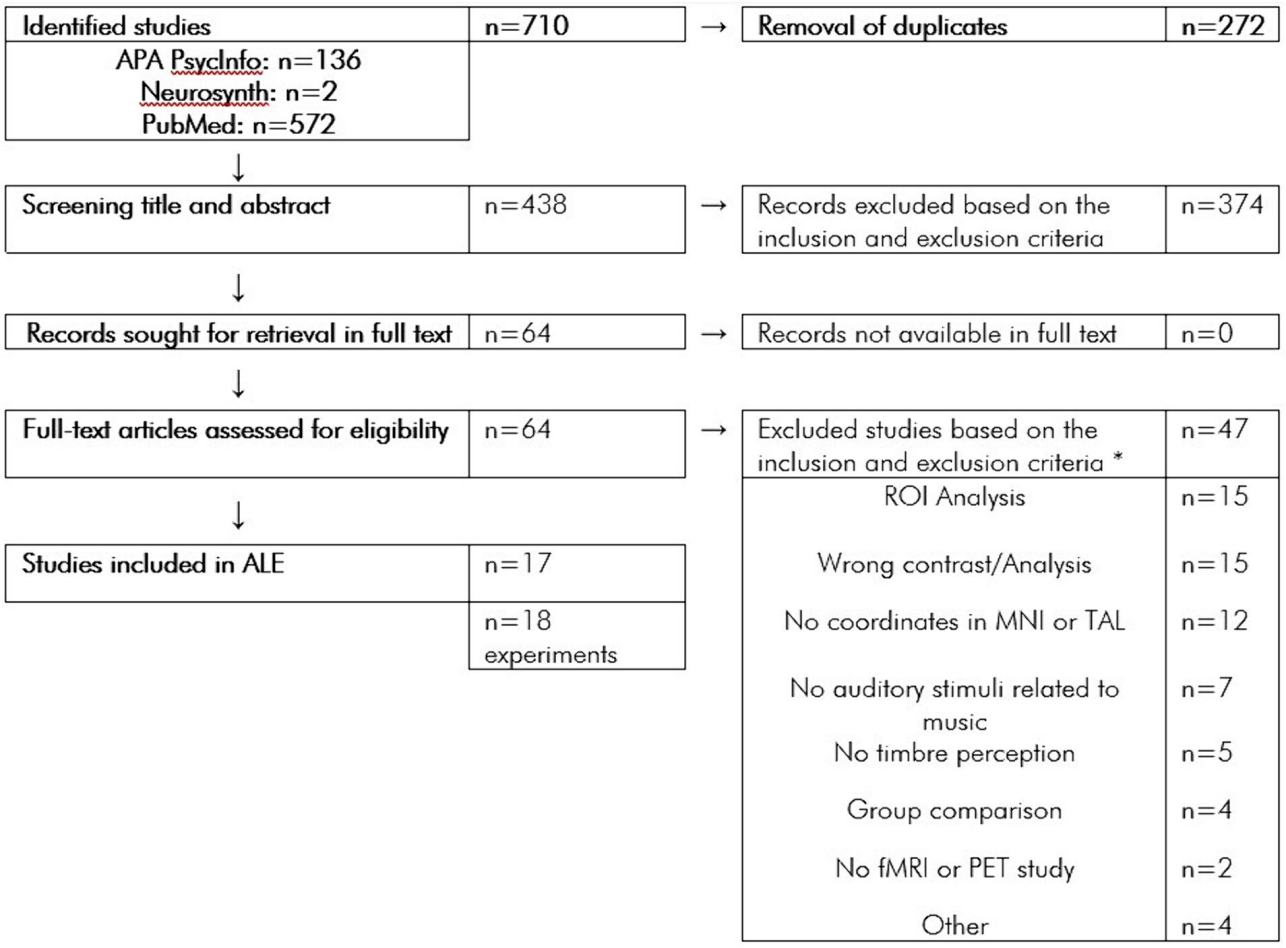
Flowchart of the article screening procedure. *n = 14 Studies were excluded for multiple reasons.

A systematic search for neuroimaging studies on musical timbre processing was carried out in the Databases PubMed, APA PsycInfo, and Neurosynth. For the search on PubMed and APA PsycInfo, we used the following search terms: “[timbre OR timber^1^ OR timbral] & [fmri OR pet]”; “music & [unexpected OR expectancy OR spectral OR noise OR [noise & scanner] OR drum OR percussion] & fmri”; “instrument & [different OR [music & emotion] OR [musical & voices] OR [music & different]] & fmri”; “[reverberation OR beatboxers] & [fmri].” The search returned n = 572 results on PubMed and n = 136 results on APA PsycInfo. Search using “timbre” returned n = 2 results on Neurosynth. In total, the search yielded n = 710 results. After the removal of n = 272 duplicates, n = 438 remaining records were screened for title and abstract. Of these n = 438 screened records, n = 374 records were excluded based on the inclusion and exclusion criteria. The full texts of all remaining n = 64 records were successfully retrieved. The Flowchart (Figure 1) shows the article screening procedure.

Studies were selected based on the following inclusion and exclusion criteria. Studies were included if timbre perception by healthy adults (> 18 years old) was investigated by using musical stimuli containing no words and using fMRI or PET; an analysis of the whole brain was carried out; foci were reported in Montreal Neurological Institute-Hospital (MNI) or Talairach (TAL) (Talairach and Tournoux, 1988) coordinate system; if the analyses or contrasts were reported for [timbre > baseline] or [timbre > rest] or [musical timbre > nonmusical timbre] or [attention timbre > attention other]. Studies were excluded, if either populations under the age of 18 years or populations with a disorder or disability were investigated; the analysis was a region-of-interest (ROI) or volume-of-interest (VOI) analysis; the article was a review or did otherwise not report original experimental research; contrasts or analyses reported were other than specified by the inclusion criteria; a group comparison was carried out; only functional connectivity analyses were carried out; and the task was timbre production or imagination.

Of n = 64 full-text articles assessed for eligibility according to these criteria, *n* = 47 studies were excluded for the following reasons: contrast that does not meet the inclusion criteria (*n* = 15); only ROI analysis was carried out (*n* = 15); no coordinates were reported in MNI or TAL (*n* = 12); no musical stimuli were used (*n* = 7); no timbre perception task were used (*n* = 5); they were non-fMRI or non-PET study (*n* = 2); only group comparison was carried out (*n* = 4); they were review article (*n* = 1); they did not study healthy adult population (*n* = 1); the analysis did not cover the whole brain (*n* = 1); and only functional connectivity analysis was carried out (*n* = 1).

From the remaining 17 studies, *n* = 18 experiments were included in the meta-analysis. Concerning the selection of contrasts, we aimed to include contrasts in a way that broadly encompasses the various aspects of processing musical timbre. These include the processing of basic perceptual features like brightness or roughness, musical sequences based on timbre, and categorical information from isolated sounds such as stimulus identification and recognition. Due to the limited number of studies investigating timbre and the heterogeneity of the contrasts, it was not possible to focus on one specific contrast.

For studies that compared passive listening to sounds with musical timbre and passive listening to sounds with a nonmusical timbre, we chose the contrast [musical timbre > non-musical timbre]. This contrast should include the activations specific to processing musical instrument identity, which is a central aspect of musical timbre processing.

For studies that compared manipulations of timbre with other parameters, we chose the contrasts between timbre and a baseline containing sounds without any timbre manipulation [timbre > baseline]. Examples include deviations in timbre versus deviations in other musical parameters or listening to complex musical timbres versus more sine-like timbres. These contrasts should reflect musical timbre processing because activations associated with the processing of loudness and tonal or rhythmic features as well as general auditory processing should be subtracted.

For studies that compared listening to musical instrument timbres of isolated sounds to resting baselines or visual and motor control baselines, we chose the contrast [timbre > rest] and [timbre > visual & motor baseline]. These contrasts should primarily reflect spectral feature processing due to the lack of tonal or rhythmic context in single sounds. Because these contrasts encompass activations from the general processing of basic timbral features in musical sounds, they allow our analysis to also capture the correlates of processing foundational aspects of musical timbre, like brightness or roughness, otherwise lost.

For studies that compared the tracking of deviations in a sequence of timbres with passive listening and a resting baseline, we chose the contrast [timbre > rest] if no contrast against a sequence of nonmusical timbres was available. This contrast should include the activations associated with sequential and structural processing of musical timbre. We did not choose the contrast [tracking deviations > listening] because it emphasizes the activations associated with paying attention to deviations and weakens those associated with the sequential processing of musical timbre.

For studies that compared listening while attending to a musical timbre and listening while not attending to musical timbre, we chose the contrast [attention timbre > attention other]. We argue that due to the nature of the contrast and the presence of musical stimuli in both conditions, general auditory activations are ruled out, activations because of processing a class of specific stimuli (that is, music) are ruled out, and correlates of general attention-paying or resources used for attentional focus are ruled out. What should remain of these contrasts are activations specific to the attentive tracking of timbre.

For studies that correlated acoustical features with brain activations during music listening, we included timbral complexity regressors. Timbral complexity is based on measures of the Wiener entropy of the spectrum, with the lowest values (no complexity) being found for sine-like tones, while more complex spectral information results in high values (Alluri and Toiviainen, 2010). We chose this regressor instead of the other common timbre-related regressors such as those targeting the perceived “fullness” (spectral fluctuations in lower bands of the spectrum), “activity” (roughness and flux in the highest areas of the spectrum), or “brightness” (spectral centroid) (Alluri and Toiviainen, 2010) because these regressors are constrained to very specific sub-aspects of timbre processing compared to the timbral complexity regressor.

Instrumental sounds were chosen over sung stimuli to minimize the influence of activations reflecting voice processing possibly shared with the domain of speech. Only one contrast per experiment was selected to control for multiple comparisons (Müller et al., 2018). Characteristics of the included 18 experiments (n = 338 Participants (159 female), mean age = 24,51 years) are shown in Table 1. The neuroimaging method comprised fMRI (*n* = 17 experiments) and PET (*n* = 1 experiment). The task varied, with *n* = 7 experiments using passive listening, *n* = 5 using musical timbre identification, *n* = 2 using detection of musical timbral deviances, *n* = 2 using listening and covert recitation of musical timbres, *n* = 1 musical timbre discrimination, and *n* = 1 on careful listening. Stimuli from Tsai et al. (2010), and Wallmark et al. (2018a,b) partly comprised sung vocal timbres (in both studies, 25% of stimuli were sung vocal sounds without text). All other stimuli consisted of non-vocal musical timbres. The foci included both cortical and subcortical activations.

**Table 1:** List of the experiments included in the meta-analysis.

Reference	N*	Task	Stimuli	Contrast/Analysis
Alluri et al. (2012)	11	Passive listening	Music	Timbral complexity regressor
Angulo-Perkins et al. (2014)	53	Passive listening	Instrument tones	Musical timbre > vocalizations
Bogert et al. (2016)	56	Identifying sound source	Music	Identify Timbre > identify Emotion
Halpern et al. (2004)	10	Passive listening	Instrument tones	Timbre > silence + visual control
Janata et al. (2002)**, Exp. I	12	Tracking timbre	Music	Attend to timbre > passive listening
Janata et al. (2002), Exp. II	15	Tracking deviation	Music	Timbre > silence
Koelsch et al. (2002)	10	Tracking deviation	Chord sequences	Timbre deviation > no deviation
Menon et al. (2002)	10	Passive listening	Instrumental melodies	Complex timbre > simple timbre
Osnes et al. (2012)	18	Identifying sound source	Instrument & sung sounds	Expecting musical timbre > neutral task
Platel et al. (1997)	6	Discrimination	Instrumental melodies	Timbre difference > other difference
Sturm et al. (2011)	24	Identifying sound source	Instrument tones & animal sounds	Timbre > visual & motor control
Tervaniemi et al. (2006)	17	Identifying sound source	Single sounds	Musical Timbre > phonemes
Toiviainen et al. (2014)	15	Listening carefully	Music	Timbral complexity regressor
Tsai et al. (2010)	12	Listening and covert recitation	Sound sequences	Musical Timbre > vocal timbre
Tsai et al. (2012)	15	Listening and covert recitation	Sound sequences	Musical timbre > pink noise
Wallmark et al. (2018a)	15	Passive listening	Instrument tones	Timbre > silence
Wallmark et al. (2018b)	15	Passive listening	Instrument tones	Timbre > silence
Whitehead and Armony (2018)	24	Passive listening	Music	Timbre > Speech

*Participants. **Janata et al. (2002) conducted two experiments with different tasks and different participants each. The term “music”, here, refers to pieces with more than one voice playing at once, as opposed to studies using monophonic melodies. “Instrument tones” refer to single, isolated sounds from musical instruments.

We conducted an activation likelihood estimation (ALE) meta-analysis. ALE is a coordinate-based statistical method that allows identifying brain areas of consistent activation across neuroimaging studies (Turkeltaub et al., 2002). The ALE was computed using GingerALE v3.0.2 (brainmap.org/ale). First, TAL coordinates were converted to the MNI system (SPM) or, if indicated, MNI (other) by the built-in conversion algorithm (Fox et al., 2013). For the calculation of the activation likelihood, a cluster-forming threshold of *p* < 0.001 with 5,000 permutations was chosen (Eickhoff et al., 2012). A cluster-wise family-wise error correction was performed using a threshold of *p* < 0.05 (Eickhoff et al., 2012; Müller et al., 2018).

## 3 Results

**Figure 2:**
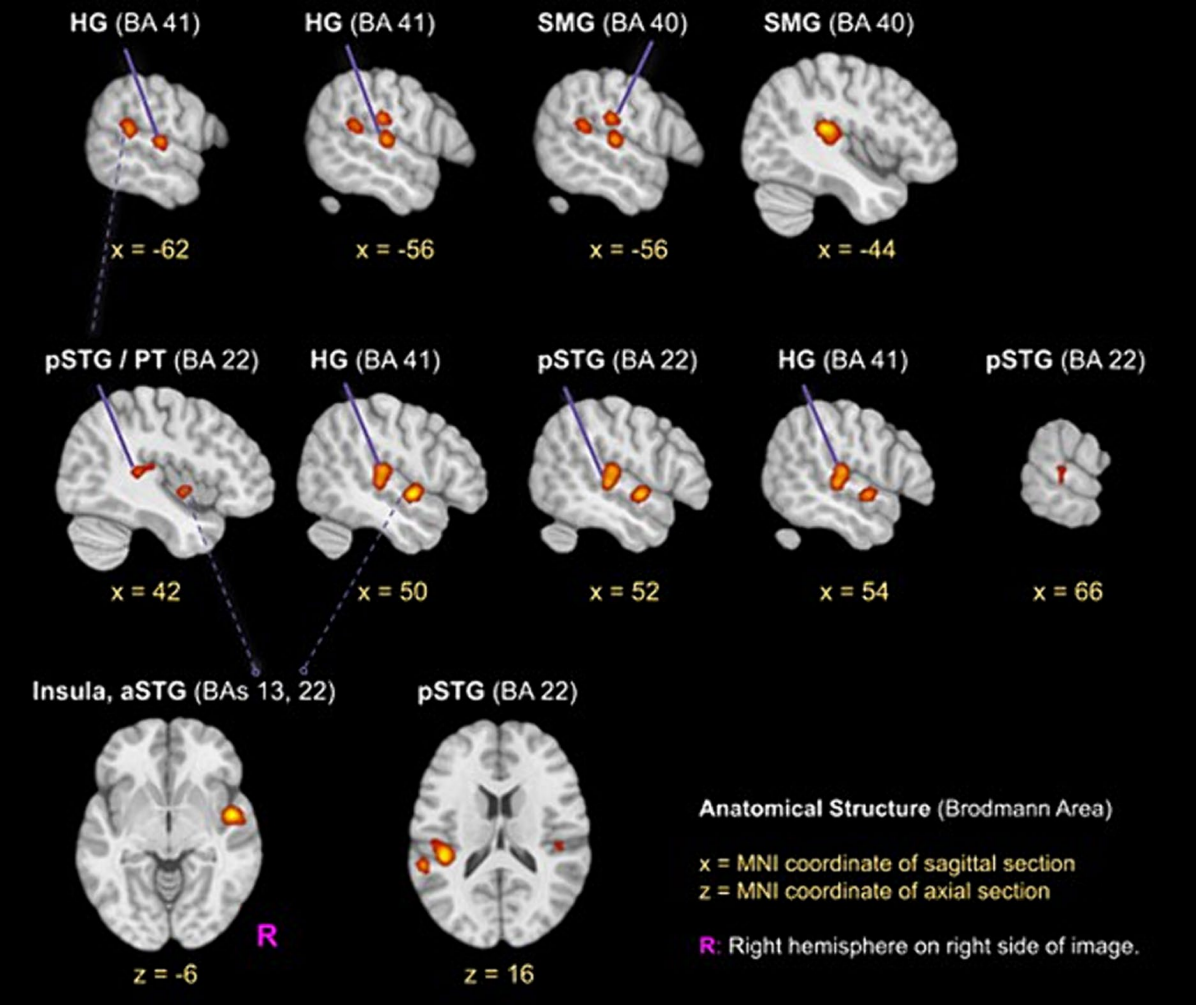
ALE clusters for the musical timbre meta-analysis Template: ICBM 2009c Nonlinear Asymmetric (T1w) with ICBM 2009c brain mask (Fonov et al., 2009, 2011). The first and second horizontal row, respectively, show peaks of clusters one and two in the pSTG, HG and SMG, the third row shows the cluster three (*z* = −6) in the right aSTG/Insula and cluster four in the left pSTG. Labels for peaks in off-white color. aSTG, anterior superior temporal gyrus; BA, Brodmann area; HG, Heschl’s Gyrus (Transverse temporal gyrus); pSTG, posterior superior temporal gyrus; SMG, supramarginal gyrus.

The ALE meta-analysis identified four clusters of consistent activation during musical timbre processing (Figure 2). Musical timbre processing consistently activated the bilateral posterior temporal lobes, including pSTG, PT, and HG. In the parietal lobe, inferior areas of the SMG were reliably activated, with more pronounced activations in the left hemisphere. In both hemispheres, posterior portions of the insula were activated. Finally, we also identified a right-hemispheric cluster covering the anterior insula and the aSTG/PP. Table 2 lists the peak coordinates of the four obtained clusters in MNI space.

**Table 2:** Peak coordinates of the clusters found by the ALE meta-analysis in MNI.

		BA	*x*	*y*	*z*	ALE	Cluster size (mm^3^)
1	Left SMG	40	−44	−34	16	0.022	4.640
	Left HG	41	−56	−20	8	0.017	
	Left HG	41	−62	−18	6	0.017	
	Left SMG	40	−56	−24	24	0.016	
2	Right pSTG/PT	22	52	−24	2	0.018	3,128
	Right HG (SMG)	41 (40)	54	−22	10	0.018	
	Right HG	41	50	−24	10	0.018	
	Right pSTG	22	42	−34	10	0.013	
	Right pSTG (TPJ)	22 (42)	66	−28	4	0.010	
3	Right ant. Insula (aSTG/PP)	13 (22)	48	−2	−6	0.023	1.696
4	Left pSTG/PT	22	−60	−40	16	0.016	864

Identified gray-matter areas which were directly adjacent to the peak of the respective cluster are noted in brackets. ant., anterior; aSTG, anterior superior temporal gyrus; HG, Heschl’s Gyrus; PoCG, Postcentral Gyrus; post., posterior; pSTG, posterior superior temporal gyrus; PT, planum temporale; PP, planum polare; SMG, supramarginal gyrus. Coordinates were assigned to Brodmann areas using the software BioImage Suite (https://bioimagesuiteweb.github.io/webapp/mni2tal.html; Lacadie et al., 2008).

Cluster 1 had its maximum in inferior parts of the SMG (BA40) in the left hemisphere. It extended ventrally into the pSTG and PT (BA 22). Anteroventrally, the cluster extended into the primary and secondary auditory cortex on HG and bordering areas (BA 41 and BA 42). Cluster 1 also covered parts of the posterior Insula (BA 13). Cluster 1 included foci from 11 experiments, of which four involved musical sequence processing such as tracking of a timbre (Janata et al., 2002, Exp. I) or timbral deviations (Janata et al., 2002, Exp. II; Koelsch et al., 2002), and attentively listening to a sequence of timbres and covert reciting (Tsai et al., 2012). One involved listening to a complex musical stimulus, using a timbral complexity regressor (Alluri et al., 2012). The other six experiments involved processing timbre from single sounds that were passively listened to (Halpern et al., 2004; Wallmark et al., 2018a,b) or that were the basis for a sound source identification task (Tervaniemi et al., 2006; Sturm et al., 2011; Osnes et al., 2012).

Cluster 2 was located in the right hemisphere, had its maximum on the pSTG (BA 22), and extended anteriorly into the HG and adjacent pSTG (BA 41 & BA 42), medially into the posterior Insula (BA 13), and dorsally into the SMG (BA 40). Cluster 2 included foci from 9 experiments involving musical sequences that contained timbre deviations (Janata et al., 2002), Exp. II; Koelsch et al., 2002), timbre sequences based on percussive sounds (Tsai et al., 2010, 2012), and isolated musical sounds (Halpern et al., 2004; Tervaniemi et al., 2006; Osnes et al., 2012; Whitehead and Armony, 2018; Wallmark et al., 2018a,b).

Cluster 3 was located in the right hemisphere anterior to Cluster 2, covering inferior anterior parts of the insula (BA 13), where it had its maximum. Laterally, it extended into the left aSTG (BA 22). This anteroventral cluster included foci from 7 experiments, all of which involved passive listening tasks: Passive listening to musical timbres from single sounds (Menon et al., 2002; Angulo-Perkins et al., 2014; Whitehead and Armony, 2018; Wallmark et al., 2018a,b), a timbral complexity regressor from a passive listening task (Alluri et al., 2012), or the passive listening to timbre deviations (Koelsch et al., 2002).

Cluster 4 was located in the left hemisphere with its maximum in caudal parts of the STG near the temporoparietal junction. The majority of cluster 4 covered ventral parts of the posterior insula (BA 13). It included foci from 5 experiments, of which two involved passive listening to the musical timbres (Halpern et al., 2004; Wallmark et al., 2018b) and three involved attending sequences with timbre deviations (Janata et al., 2002), Exp. II; Koelsch et al., 2002) or attentive listening to timbre sequences (Janata et al., 2002), Exp. I).

Although subcortical foci were included, the four clusters did not cover subcortical structures.

## 4 Discussion

### 4.1 Comparison with the previous studies

The goal of this study was to identify areas of consistent activation during the processing of musical timbre through an ALE meta-analysis. We identified clusters in the bilateral pSTG, SMG, and the right aSTG and anterior insula.

Our results partially confirm expectations based on the literature and the findings from two earlier ALE meta-analyses on musical timbre processing. Our analysis confirms the involvement of the bilateral STG, including primary AC and PT (Janata, 2015) and in the right IPL (Criscuolo et al., 2021), while the involvement of the cerebellum (Janata, 2015) and the IFG (Criscuolo et al., 2021; Wei et al., 2022) could not be confirmed. The cerebellum is primarily involved in timing and rhythmic processing and is currently not thought to be involved in timbre processing (Evers, 2023). The IFG is foremost associated with hierarchical/syntactic processing in music (Musso et al., 2015; Asano et al., 2021), which most studies included in our analysis did not test. Moreover, we identified an additional involvement of bilateral temporoparietal regions, the bilateral posterior insular cortex, and the right anterior insular cortex in musical timbre processing, which was not identified by previous analyses (Janata, 2015; Criscuolo et al., 2021) nor considered by reviews (Town and Bizley, 2013; Alluri and Kadiri, 2019; Wei et al., 2022). While the IPL is central for processing sequential information (Rauschecker, 2014), the posterior and anterior portions of the insula are involved in sound detection, sensory and motor processing, temporal processing and sequencing (Bamiou et al., 2003) and emotional processing (Gu et al., 2013; Koelsch, 2018), respectively. Compared to previous meta-analyses, we included far more contrasts using musical sequences, which possibly led to the pronounced involvement of the IPL and the posterior insula. Also, many of our contrasts used natural music presumably containing richer emotional and sensorimotor information than isolated sounds, which may explain the activations in the right anterior insula (for discussions, see Asano et al., 2022b) and the bilateral posterior insula.

### 4.2 Timbre processing in the dual streams

**Figure 3:**
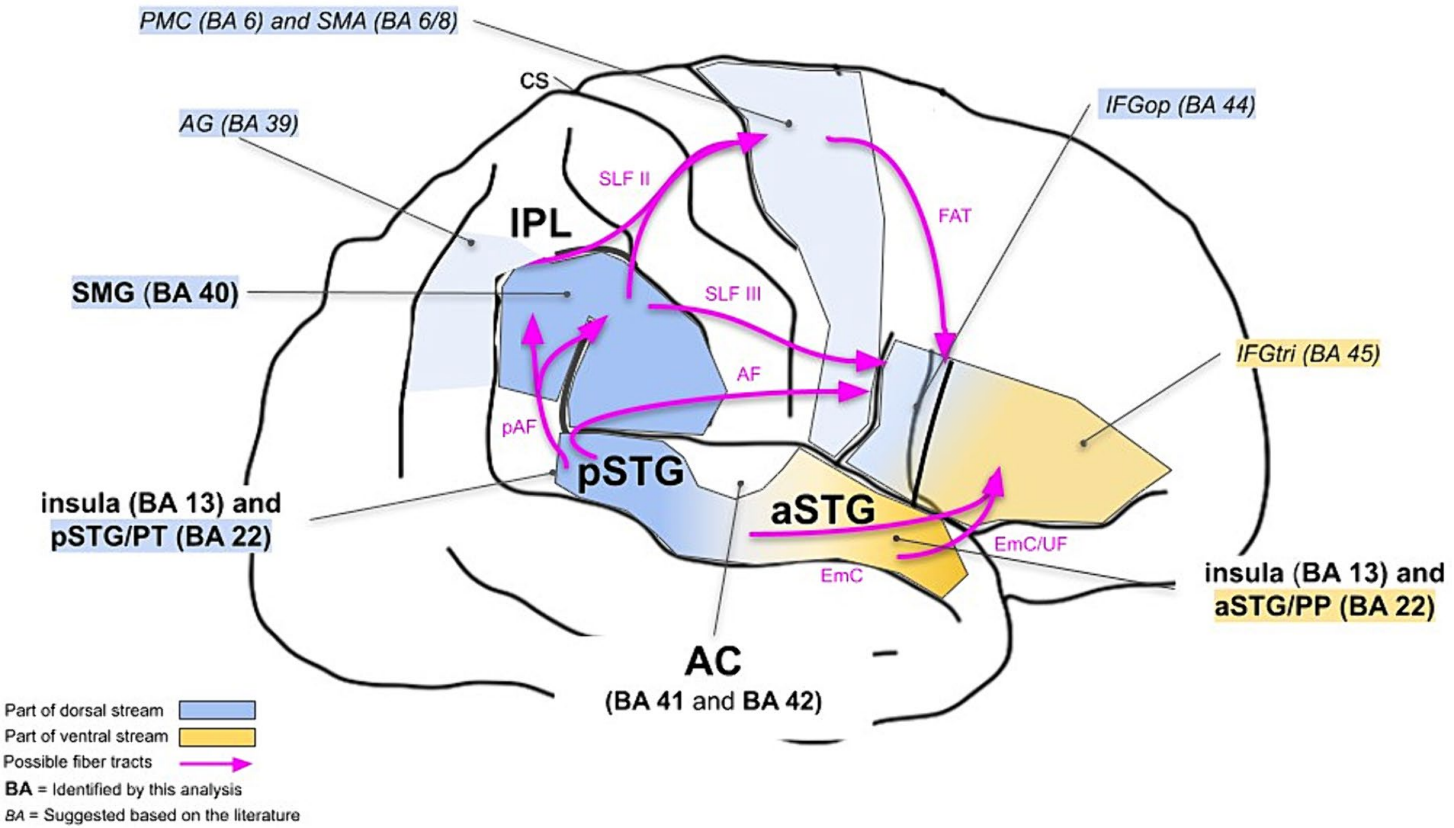
Dual-stream model for cortical processing of musical timbre. It shows the right hemisphere. Areas reported by the previous studies on cortical processing of musical timbre are referred to in italic letters. The areas referred to in bold were identified in the present meta-analysis. AF, Arcuate Fasciculus; AG, angular gyrus; CS, central sulcus; EmC, Extreme Capsule; FAT, Frontal Aslant Tract; IFG, inferior frontal gyrus; pAF, posterior segment of the Arcuate Fasciculus; PMC, premotor cortex; PP, planum polare; pre-SMA, pre-supplementary motor area; PT, planum temporale; SLF, Superior Longitudinal Fasciculus; SMA, supplementary motor area; SMG, supramarginal gyrus; STG, superior temporal gyrus; UF, Uncinate Fasciculus.

We particularly aimed to investigate whether the neuronal correlates of musical timbre processing can be explained in a dual-stream model. Based on the previous research, timbre processing in the dual-stream model can be described as follows (see also Figure 3, italics).

The ventral stream areas involved in musical timbre processing are regions in the aSTG, anteroventral to the auditory cortex, which are known to mediate the recognition and identification of musical stimuli (Rauschecker, 2014; Alluri and Kadiri, 2019) and have extensive connections to regions in the IFG (Rauschecker and Scott, 2009).

The dorsal stream regions pSTG & PT, IPL and PMC & SMA, and IFG are activated by musical timbre processing in discriminating successive sounds (Warren et al., 2005; Town and Bizley, 2013; Allen et al., 2017), maintaining representations of timbres (Halpern et al., 2004) and processing sound-motor relationships underlying timbre (Margulis et al., 2009; Tsai et al., 2010, 2012; Lévêque and Schön, 2015; Krishnan et al., 2018; Wallmark et al., 2018a,b), and processing structural properties of musical sequences based on timbre (Koelsch et al., 2002; Reiterer et al., 2008), respectively.

The results of our meta-analysis highlight the involvement of the ventral stream areas and some parts of the dorsal stream areas, and complement it with bilateral activations in the insular cortex (see also Figure 3, boldface).

Cluster 1 and 2 involve dorsal stream areas such as the bilateral pSTG (BA 22) and IPL (SMG, BA 40). The experiments that investigate tracking a timbre or timbral deviations in a sequence and the attentive listening to a sequence of timbres contributed to those clusters, suggesting that these areas are involved in sequential processing based on timbre. However, looking into the details, pSTG and IPL seem to have a division of labor in sequence processing. Compared to cluster 1, which has its peak in BA 40 and partly extended into BA 22, a greater number of studies involving isolated sounds contributed to cluster 2, which has its peak in BA 22 and extends partly into BA 40. We suggest that this is because BA 22 is more associated with processing smaller units in sequencing, such as segregating incoming auditory information into spectrotemporal patterns that correspond to distinct perceptual units (Griffiths and Warren, 2002), while the IPL is more associated with processing larger units in sequencing because it maintains representations of sounds (Rauschecker, 2011, 2014).

Cluster 3 involves the right aSTG as a ventral stream area. The experiments which investigate the passive listening to isolated musical sounds mainly contributed to this cluster (5/7 contrasts; Menon et al., 2002; Angulo-Perkins et al., 2014; Wallmark et al., 2018a,b; Whitehead and Armony, 2018), suggesting that this area is associated with the processing of timbral features and the extraction of emotional and categorical information based on timbre, because no tonal or rhythmic cues are provided, but only single sounds. Based on timbre-related acoustical features of single musical sounds (e.g., spectral and temporal envelope, or the spectral centroid), listeners can extract stimulus identity and emotion (McAdams et al., 2017; Ogg and Slevc, 2019), which are typical timbre-based functions of the ventral auditory stream along the aSTG (Rauschecker, 2014).

To cluster 4, which had its peak in areas of the left pSTG near the temporoparietal junction, the contrasts of Koelsch et al. (2002), Janata et al. (2002, Exp. II), and Janata et al. (2002, Exp. I) contributed and these can be interpreted as the temporal processing and sequencing based on timbre because the studies involved attending sequences with timbre deviations and attentive listening to timbre sequences. This is consistent with the involvement of the pSTG in sound detection, sound discrimination, and sequencing in general auditory processing (Griffiths and Warren, 2002; Rauschecker and Scott, 2009). The posterodorsal parts of the STG connect to other areas in the posterior temporal lobe and the parietal lobe (Makris et al., 2017; Román et al., 2022), as identified by clusters 1 and 2, that is, other areas of the dorsal stream.

Moreover, clusters 3 and 4 involved the anterior and posterior insula (BA 13), respectively. The involvement of the anterior insula is in line with recent findings from other musical domains that assume the contribution of the insula in music processing in the dual streams (Asano et al., 2022b). Musical timbre may be processed through auditory-motor interactions mediated by the insula, which connects to areas of the motor system and the limbic system because timbre not only conveys the identity of the sound source itself but also the physical and emotional state of the sound source (Wallmark et al., 2018b). Following this line of reasoning, we suggest that the consistent activation of the insula in our analysis could be explained by the sensorimotor and emotional processing of timbre sequences and potentially encoded emotional information.

Our meta-analysis did not find the motor areas of the dorsal stream (PMC/SMA, BA 6/8). A central function of BA 6 and 8 in music processing is linking perception and production (Zatorre et al., 2007). For example, BA 6 and 8 have been proposed to represent pitch sequences as motor patterns (Rauschecker, 2014) and were associated with establishing sound-action associations based on timbre (Wallmark et al., 2018b). In our analysis, two of the contrasts involved the covert recitation of perceived timbres (Tsai et al., 2010, 2012). However, the majority of contrasts did not entail tasks that emphasized processes relating sound to action.

Although our meta-analysis did not find all regions associated with the dorsal and ventral streams, previous clinical findings support the conceptualization of musical timbre processing in the dual streams. For example, Mazzucchi et al. (1982) and Peretz et al. (1994) report that patients with lesions in the middle and anterior temporal lobes (that is, ventral auditory stream areas) suffer from impaired categorization based on timbre, while the discrimination of subsequent sounds based on timbre remained intact. Mazzoni et al. (1993), on the other hand, describe a patient with lesions in posterior temporal and inferior parietal cortices (that is, areas of the dorsal auditory stream) suffering from impaired discrimination of sequences of timbres, with intact identification of sound source categories (instruments) based on timbre. We thus suggest that the dual stream model provides a useful framework for future research on musical timbre processing.

### 4.3 Further implications: domain-specificity vs. -generality?

We suggested that musical timbre processing relies on areas and networks that can be explained in a dual-stream model. Similar models were proposed for music (Zatorre et al., 2007), including tonal (Musso et al., 2015), and rhythmic (Patel and Iversen, 2014) processing, and general auditory processing (Rauschecker and Scott, 2009). Recently, it was argued that cognitive systems like music can be understood as specialized ways of using domain general information processing operations performed by regions or circuits (Asano et al., 2022a). Following this view, musical timbre and other auditory processing could rely on shared neural bases implemented in the dual streams, which may be embedded in different networks including sub-regions specialized during development and/or evolution for musical timbre processing. In this case, we should see similarity between the neuronal correlates of musical timbre processing and of other auditory functions, such as general music, rhythmic, tonal, and general auditory processing. At the same time, there should be some specialization for musical timbre processing within the dual streams.

Comparison between our meta-analysis and other meta-analyses and reviews reveals that musical timbre processing indeed involves brain areas similar to music processing in general, but also tonal and rhythmic processing and general auditory processing, yet often differs concerning the involved sub-regions.

Similarities between the brain regions involved in music processing in general as indicated by two meta-analyses on passive music listening (Gordon et al., 2018; Pando-Naude et al., 2021) and musical timbre processing concern the bilateral HG and pSTG/PT, IPL, and the aSTG. This similarity between musical timbre processing and passive music listening may have resulted from the similarity of stimulus features. For example, the results of the two meta-analyses could reflect cortical correlates of processing perceived timbre because most of included studies used stimuli with complex musical timbres (e.g., musical instrument sounds).

The brain regions activated in musical rhythm processing as indicated by a meta-analysis (Heard and Lee, 2020) and timbre processing are the bilateral pSTG and the bilateral SMG, which may result from similar types of processing such as complex sequence processing (Rauschecker and Scott, 2009; Rauschecker, 2014). Within the SMG, however, rhythmic processing involves postero-dorsal areas bordering the AG, whereas the areas involved in musical timbre processing are located more inferior and anterior. A meta-analysis of beat-based rhythmic processing (Kasdan et al., 2022) and our results do not exhibit any similarities.

Pitch processing, as reported in two recent meta-analyses on tonal processing (Asano, 2019; Asano et al., 2022b), and musical timbre processing both revealed involvement of the right anterior insula, which also might result from a shared type of processing. The right anterior insula is considered to process perceived emotion in various auditory domains (Seydell-Greenwald et al., 2020; Koelsch et al., 2021), and both tonal features and musical timbre are seen as principal ways for conveying musical emotion (Athanasopoulos et al., 2021). Tonal processing, however, involves rostral portions of the right anterior insula and the aSTG, while musical timbre processing is located more posterior and inferior.

Musical timbre processing also involves brain areas similar to general auditory processing. Consistently, all areas indicated in the current meta-analysis apart from the anterior insula are involved in general auditory processing of humans and other primates (Rauschecker and Scott, 2009). These similarities could, however, potentially arise from contributions of general auditory areas to the identified clusters. This is because some of the contrasts used in the analysis, such as rest and other non-auditory baselines, may have included these areas. To further investigate this possibility, we examined the output of the ALE analysis and conducted an additional analysis of the foci in the timbre > rest and timbre > motor/visual control baseline contrasts (see supplementary materials). We examined how much these contrasts contributed to our clusters. The extra analysis finds that these contrasts contributed to, but did not dominate the clusters of our ALE analysis.

In sum (see also Table 3), the areas involved in processing musical timbre identified in the present ALE meta-analysis are similar to the areas and networks recruited for processing information in other musical domains and for general auditory processing. As we identified largely converging neural structures for processing musical timbre and processing in other domains, we presume that musical timbre processing relies on neural resources used by other auditory domains and vice versa. In future research, however, it is still necessary to study if there are some neural specializations for each domain within those shared regions to advance the discussions on domain-specificity vs. – generality (for discussions, see Asano et al., 2022a).

**Table 3:** Areas involved in musical timbre processing (this meta-analysis) and their relation to tonality and rhythm processing, passive music listening, and general auditory processing.

Musical timbre			Other domains			
Identified location		BA	Tonality	Rhythm	Music listening	General auditory
L	SMG	40	–	(4)	–	(7)
L	HG	41	–	–	(5,6)	(7)
L	HG	41	–	–	(5,6)	(7)
L	SMG	40	–	(4)	–	(7)
R	pSTG / PT	22	–	–	–	(7)
R	HG (SMG)	41, (40)	(2), −	–, (4)	(5), −	(7)
R	HG	41	(2)	–	(5)	(7)
R	pSTG	22	–	–	(6)	(7)
R	pSTG (TPJ)	22, (42)	–	(4), (4)	–, (6)	(7)
R	Insula (aSTG/PP)	13, (22)	(1), (1, 2)	–, −	–, (6)	–, (7)
L	pSTG/PT	22	–	(4)	(6)	(7)

L, Left hemisphere; R, Right hemisphere. Shared location with results from ALE meta-analyses on tonal processing: (1) Asano et al. (2022a); (2) Asano (2019); rhythm processing: (3) Kasdan et al. (2022) (none); (4) Heard and Lee (2020); passive music listening: (5) Gordon et al. (2018); (6) Pando-Naude et al. (2021); and a review on general auditory processing: (7) Rauschecker and Scott (2009).

### 4.4 Limitations

The present study has several limitations.

First, the small number of studies included in this ALE meta-analysis poses limits to the power of this meta-analysis (Eickhoff et al., 2016; Müller et al., 2018). Due to the dearth number of published studies available for the analysis, we were able to slightly exceed the recommended minimum of 17 experiments for a reliable ALE meta-analysis (Eickhoff et al., 2016; Müller et al., 2018).

Second, tasks and contrasts greatly varied (i.e., studies were very heterogenous). That is, we pooled activations across a broad variety of processes based on timbre, resulting in a rather coarse-grained estimate of the neural correlates of musical timbre processing.

Third, we included non-auditory baseline and resting contrasts, which may have introduced activations in the AC due to general auditory processing. In 4.3, we argued that contrasts against rest and visual/motor control tasks contributed to, but did not dominate the clusters of our ALE analysis, which thus likely do not primarily reflect general auditory processing. Still, in future research, once more studies with contrasts examining musical timbre more specifically are available, it is worth doing a further meta-analysis excluding rest contrast to pursue this issue.

Fourth, the involvement of subcortical structures in musical timbre processing was not discussed because our focus was mainly put on the dual stream model of timbre processing. However, areas in the dorsal and ventral streams receive input from and project to subcortical structures, and they together implement several functions (for an overview concerning the general auditory processing, see Rauschecker, 2021). Thus, the possible interplay between the dual streams and subcortical structures for musical timbre processing should be further investigated and research on the subcortical structures should be integrated into our model in the future to provide a more complete picture.

## 5 Conclusion

This paper investigated the neural correlates of musical timbre processing through an ALE meta-analysis of 18 experiments from 17 neuroimaging studies. Musical timbre processing consistently involved activations in the bilateral auditory cortex (BAs 41, 42), bilateral postero-dorsal regions including the pSTG / PT (BA 22), the SMG (BA 40), and the posterior insula (BA 13), as well as antero-ventral regions in the right aSTG / PP (BA 22), and the right anterior insula (BA 13). Apart from the insula, these areas are associated with the dorsal and ventral auditory streams, providing evidence for dorsal components of cortical processing of musical timbre besides the well-known ventral auditory stream involvement. Thus, we proposed to frame musical timbre processing in a dual-stream model, which serves as a framework for future investigations into the neuronal processing of timbre. Moreover, similar to research on other musical domains, this model is complemented with an involvement of the insula. The results of our meta-analysis also suggest that musical timbre processing may rely on neural resources shared with other musical and non-musical auditory domains. Therefore, future music cognition research should elucidate timbre processing in the dual streams, not least through the examination of the relationships between the neural bases of processing musical timbre and of processing other essential features of music.

## Data availability statement

The original contributions presented in the study are included in the article/supplementary material, further inquiries can be directed to the corresponding author.

## Author contributions

OB: Conceptualization, Investigation, Methodology, Visualization, Writing – original draft. RA: Validation, Writing – review & editing.

## Footnotes

^1^The term “timber” was searched on *PubMed* only because the search for “timber” in *APA PsycInfo* yielded a plethora of articles related to the study of wood, materials research, to other fields not concerned with music at all, and a researcher named “Timbers.”
